# The perception of smile attractiveness to altered vertical position of maxillary anteriors by various groups

**DOI:** 10.1097/MD.0000000000028660

**Published:** 2022-03-04

**Authors:** Walaa A. Babeer, Zuhair T. Bakhsh, Zuhair S. Natto

**Affiliations:** a Department of Prosthodontics, Faculty of Dentistry, King Abdulaziz University, Jeddah, Saudi Arabia; b Private Practice, Orchid Dental, Jeddah, Saudi Arabia; c Department of Dental Public Health, Faculty of Dentistry, King Abdulaziz University, Jeddah, Saudi Arabia.

**Keywords:** esthetics, facial expression, maxillary teeth, perception, smiling

## Abstract

There is a gap in research about the differences in smile attractiveness. The problem the study addresses is how the vertical canine and incisor position affect smile attractiveness. The aim of this study was to assess the perception of the smile attractiveness between Saudi laypersons, orthodontists, non-orthodontist, and various dental students levels, and to determine how the canine and incisor vertical positions affect the attractiveness of smile. The study is a cross-sectional survey and was conducted at King Abdulaziz University, Jeddah, Saudi Arabia. Six groups of subjects participated in the study: Orthodontic residents (n = 31), prosthodontic, restorative, periodontics specialties residents specialties residents (n = 30), interns (n = 31), fifth year students (n = 41), 6th year students (n = 39), and laypeople (n = 39). Participants were asked to rate the attractiveness of a smile of a female subject photographed using a Minolta DiMage 7i digital camera. The image had been manipulated to produce 2 sets of images; 1 to modify the incisors and 1 to change the canines. The subjects were asked to choose the most and least attractive picture. For the best incisor positions, only the laypeople and prosthodontics liked the original picture, the rest liked +0.5 mm which accentuate the smile curve and make it follow the lower lip line. For the worst incisor position, all groups did not prefer the minus 1.5 reversed smile. For the best canine vertical position, all groups preferred the original position where canine was at the level of the incisal plane. For the worst canine position, they all disliked the minus 1.5 reversed smile. Results confirmed past findings that orthodontists are in general more critical about smile attractiveness than laypersons, but just like other dental specialists. The findings can be used in the esthetic dentistry field, but further research on the study population based on other dental design parameters remain necessary.

## 1 Introduction

An attractive smile has been shown to play an essential factor in social acceptance.^[1–3]^ During social interaction, the eyes and mouth provide the primary source of attention.^[4,5]^ The mouth, or the smile, therefore, plays a vital role in facial expression, appearance, and attractiveness.^[5,6]^

Smile analysis and smile design through the years have become crucial in orthodontic and prosthodontics practice, especially for diagnosis and treatment planning.^[7]^ Outside these dental practices, laypeople have become more critical of their smiles. Because of these refined esthetic expectations, demand for dental treatment changed from mere function to esthetics.^[8]^

Several researchers investigated the impact of various parameters of a smile on its attractiveness. These included buccal corridors, arch widths, smile arc, midline deviations, incisal inclinations, asymmetries, gingival displays, anterior proportions, and vertical position of the incisors.^[9–12]^ A range of acceptable deviations exists and laypeople are generally more tolerant of a broader range of variations.^[13]^

Attractive anterior teeth with proper size and shape were established as some of the most influential factors that contribute to an attractive smile in majority of the dental health-care professionals, whether in the orthodontic, operative, and prosthodontic management fields.^[14]^ The design of esthetic smile contrary to what ordinary people may think, is in reality, a very complicated process that necessitates a multidisciplinary approach. The popularity of designing a naturally attractive smile has literally transformed into an architectural blueprint utilized in esthetic dentistry to obtain optimal esthetic results.^[15]^

Smile attractiveness of the appearance of anterior teeth is one of the most crucial aspects of dental and facial esthetics. As such, those in the fields, regardless of their specializations, took time to analyze dental and gingival display.^[16]^ Consequently, their perceptions of dentofacial esthetics can differ from the perceptions of the laypersons. These varying views, however, need to be considered during orthodontic and prosthodontic treatment planning.^[17]^

The vertical relationship of the maxillary anterior teeth is one of the most important aspects not only for achieving proper esthetics but also for function. Their position influences aspects such as Incisal guidance, canine guidance, smile line, the setting of dentures, and anterior restorations. It is because of these influencing factors that slightly different vertical position preferences for the anterior maxillary teeth exist among specialties.^[18,19]^ In prosthodontics treatment planning, patients demand esthetic and determine the anterior teeth position and inclinations. Incisal edge position, midline, and how the anterior upper and lower teeth guide the occlusion are all factors to be considered at the beginning of prosthetic patients’ rehabilitation. In addition, the position of maxillary central incisors will dictate the positions of lateral incisors, canine, and premolars.^[20]^

In general, an attractive smile is one where the incisal edges of the upper anterior teeth follow the curvature of the lower lip while smiling.^[21,22]^ According to the McLaughlin Bennett Trevisi system, the tip of the canines should be at the level of the central incisor edge.^[23]^ Kalanga, on the other hand, recommended the maxillary canine tip to be 1 mm below the level of the central incisor.^[24]^

Studies on the vertical position of the incisors have mostly looked at gingival margin discrepancies between the incisors and canines.^[25,26]^ Only a few studies have looked at the vertical incisal edge discrepancies between the teeth.^[27–29]^ Further, some studies looked at the effect of altering the vertical canine position, the impact of combined central and lateral incisor vertical position relative to the canine, or the symmetry of lateral incisors edge position.^[30]^ The relative position of the lateral incisor in relation to the central incisor was 0 to 2 mm.^[31,32]^

In a world continuously changing, the esthetic parameters are subjected to change too. Updates on perceived esthetic considerations are therefore necessary. This study is designed to focus on Saudi population preferences and if they match the findings done on Western populations.

## 2 Material and methods

This is a cross-sectional survey study involving the use of a smiling picture modified to change the vertical position of the incisors and canine. The study was conducted at King Abdulaziz University, Jeddah, Saudi Arabia, and approved by the research ethics committee at the Faculty of Dentistry (#219-02-21). This study followed STROBE guidelines. A series of pictures were obtained and shown to subjects, who rated their attractiveness.

### 2.1 Photographs

The smile of a female subject was photographed using a Minolta DiMage 7i digital camera (Konica Minolta, Tokyo, Japan) with a ruler in the image for scale. The smile was posed with the mouth opened wide enough to remove focus from the mandibular teeth. The upper lip covered the gingival margins. The image was retouched using Adobe Photoshop 9.0 (Adobe Systems, San Jose, CA) for symmetry and to remove any irregularities to reduce distracting and confounding variables.

The image was then manipulated to modify the incisors or to change the canines. In the incisor set, the upper central and lateral were moved together vertically in 0.5 mm increments, both gingival and incisal. This resulted in a total of 7 images. The same process was repeated for the canine alone, resulting in another set of 7 images. The final set of pictures contained 15 images; 7 modified incisors, 7 modified canines, and the original image. Further, to ensure symmetry of the right and left sides of the image, 1 side of the arch was modified and then mirror imaged and combined to produce identical right and left sides. Photoshop was used to remove any irregularities and duplicate scales.

### 2.2 Subjects

Six groups of subjects were asked to participate. Orthodontic residents (n = 31), other specialties residents such as operative, prosthodontics, and periodontics (n = 30), interns (n = 31), fifth year students (n = 41), 6th year students (n = 39), and laypeople (n = 39).

### 2.3 Survey instrument

Each participant received 2 papers. The first page explained the aim of the study and that participation is voluntary. It also asked about the gender of the participant. The second page was the scoring sheet. A computer laptop was used to show participants images on a screen with a black background in a PowerPoint presentation. Image size as viewed on the screen, was identical to the actual size of the teeth. Participants were asked to view 1 image after the other and rate the attractiveness of each image on a 100 mm visual analog scale marked at 5 mm increments given to them on the survey paper. The scale was labeled at both ends with the extremes from “least attractive” near the zero on the left to “most attractive” near the right side.

All images were randomly sequenced in the presentation. One incisor image (–1.5 mm) and 1 canine image (+1 mm) were duplicated to check for reliability. Further, the original and incisor images (except for the +1.5 mm) were placed in 1 slide, and the subjects were asked to select the most and least attractive picture in the set. The same was repeated with the canine images. The score for each image was calculated using a ruler to measure the location of the mark on the scale. All data was entered first into excel.

A 100-mm visual analog scale appeared under each image in the questionnaire and was used for the ratings. It was labeled at both ends according to extremes of attractiveness, from “least attractive” near zero on the left to “most attractive” near 100 mm on the right. Each rater marked a point along the scale according to his or her perception of dental esthetics. Each rating was measured in millimeters.

**Figure 1:**
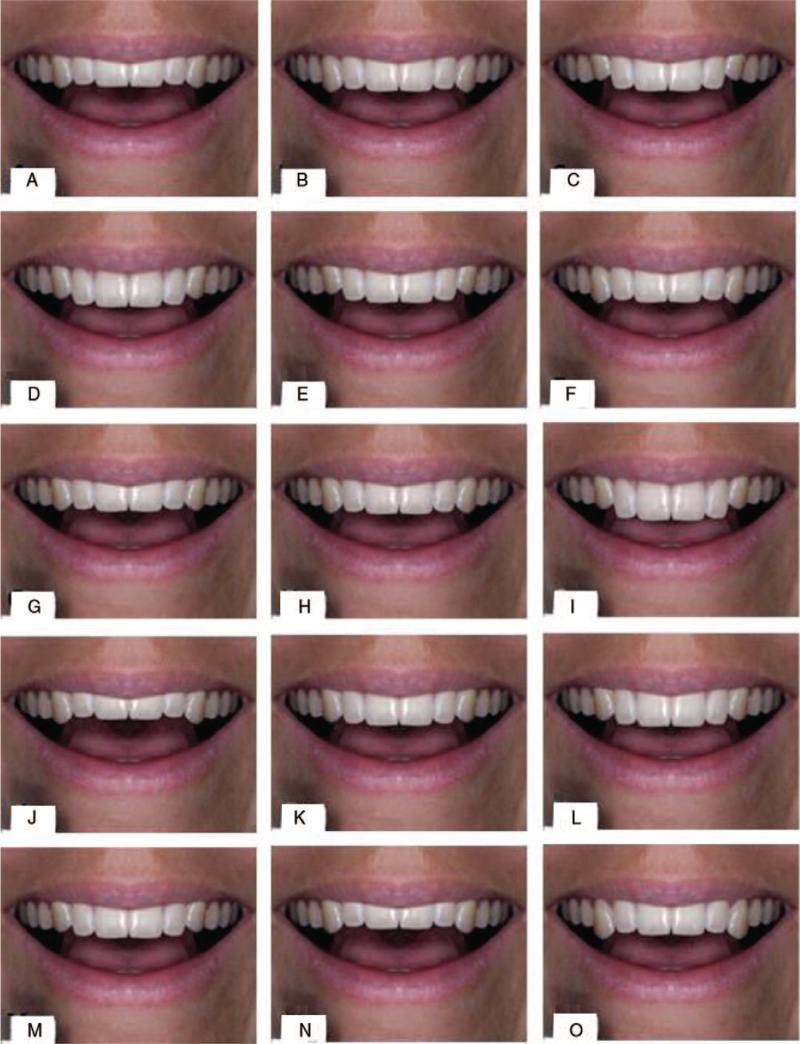
Level of the maxillary incisors or canine were created incrementally; A: incisors –1, B: canine +1, C: canine –1.5, D: incisors +1, E: canine –1, F: canine +1, G: incisors –0.5, H: canine +0.5, I: incisors +1.5, J: incisors –1.5, K: original, L: incisors +0.5, M: canine –0.5, N: incisors –1.5, O: canine +1.5.

The images were then imported into Microsoft PowerPoint (Microsoft, Redmond, WA) for slide projection. Frontal facial photographs of 1 female subject were altered using Adobe Photoshop 8.0. The judges evaluated these 15 images: 7 images with different vertical positions of canine in relation to the frontal occlusal plane and 7 with a different vertical position of the incisors in relation to the frontal occlusal plane, and 1 original image with ideal esthetic parameters. Each picture was altered with photoshop to change the height of the incisors and canines at 0.5 mm increments. The pictures were coded and arranged in a non-organized manner on purpose. Where I refers to Incisors, C refers to canine, Prefers to plus, and M refers to minus. Incisors –1, canine +1, canine –1.5, incisors +1, :canine –1, canine +1, incisors –0.5, canine +0.5, incisors +1.5, incisors –1.5 original (Orig were incisors set at zero), incisors +0.5 (IP +0.5), canine –0.5, incisors –1.5 (Fig. 1).

### 2.4 Sample size

A pilot and convenience was used in this study. All participants within the study time frame were included in the study.

### 2.5 Statistical analysis

One-way repeated analysis of variance tests were performed within each group to evaluate the deviation. Then several schafeepost-hoc comparisons between each level of variation were used to determine the level of discrimination between esthetic and less esthetic in each group. Moreover, 2-way repeated analysis of variance were conducted on each type of dental discrepancy. Statistical analysis was performed using a statistical software program (SAS, version 9.3, SAS Institute Inc, Cary, NC). The level of significance was set at *P* < .05.

## 3 Results

Threshold levels regarding incisor and canine height position were between 0.5 and 1.5 mm. It was lower for 5th and 6th-year dental students (0.5 to 1.0 mm), and higher for all other specialists (1.0 to 1.5 mm) (see Table 1).

**Table 1:** Threshold levels of significant difference (mm).

	Incisor height position	Canine height position
5th year dental students	0.5-1.0	0.5-1.0
6th year dental students	0.5-1.0	1.0
Interns	1.0	1.0
Lay people	1.0	0.5-1.0
Orthodontist	1.0	1.0
All other specialists	1.0-1.5	1.0-1.5

**Figure 2:**
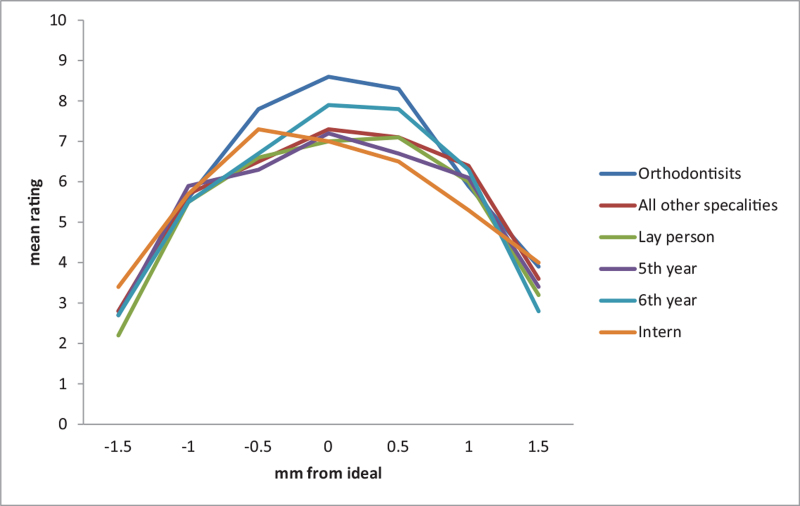
Graphical presentation of results between groups in Incisor height position.

**Figure 3:**
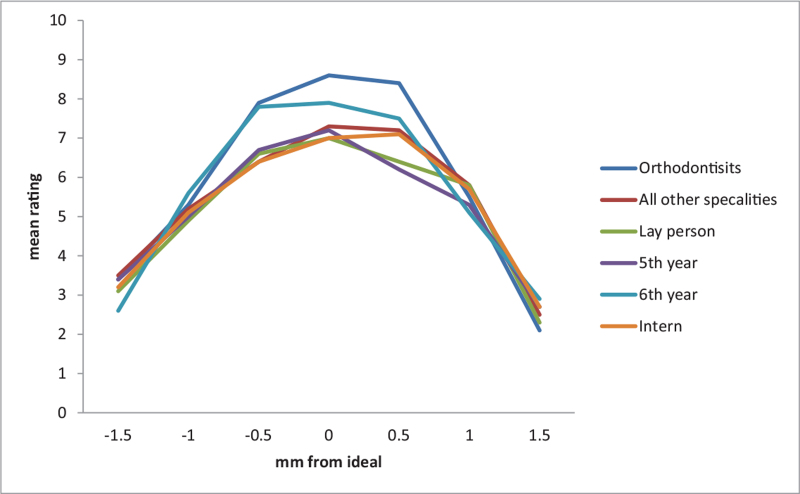
Graphical presentation of results between groups in Canine height position.

Although the best and worst incisor positions were not the same within each group and statistically significant (*P* < .0001), the positions were almost the same between all groups and (original to +0.5 in best and –1.5 in worst), and not statistically significant (*P* = .851) (see Table 2). Moreover, we have the same pattern with the canine position which was the same between groups (original for best and –1.5 for worst). However, the positions were not the same within each group (*P* = 1.000) (see Table 3). The best incisor position is between –0.5 to +0.5 in all groups. The more deviated from this range, the worst the incisor position will be expected (Fig. 2). The same pattern with the canine position. The best canine position is between –0.5 to +0.5 in all groups. The more deviated from this range; the worst the canine position will be expected (Fig. 3).

**Table 2:** Comparison of best and worst incisor position among different groups.

Group	Best incisor position	Worst incisor position	*P* value
All groups	+0.5	–1.5	.851^∗∗^
5th year dental students	+0.5	–1.5	<.0001^∗^
6th year dental students	+0.5	–1.5	<.0001^∗^
Interns	+0.5	–1.5	<.0001^∗^
Lay people	Original	–1.5	<.0001^∗^
Orthodontists	Original	–1.5	<.0001^∗^
All other specialists	+0.5	–1.5	<.0001^∗^

∗ *P* value < .05 and within each group.

∗∗ *P* value between groups.

**Table 3:** Comparison of best and worst canine position among different groups.

	Best canine position	Worst canine position	*P* value
All groups	Original	–1.5	1.000^∗∗^
5th year dental students	Original	–1.5	<.0001^∗^
6th year dental students	Original	–1.5	<.0001^∗^
Interns	Original	–1.5	<.0001^∗^
Lay people	Original	–1.5	<.0001^∗^
Orthodontist	Original/+0.5	–1.5	<.0001^∗^
All other specialists	Original	–1.5	<.0001^∗^

∗ *P* value < .05 and within each group.

∗∗ *P* value between groups.

## 4 Discussion

The effects of gingival displays on smile attractiveness is well documented in the literature. Past findings already established that orthodontists would be more critical than laypersons when dental disharmony is small. If the gingival margin discrepancy is only 2 mm between the central and lateral incisors, other dental specialists and laypersons, in general, will no longer be bothered by it nor consider their smile unesthetic.^[33]^ Previously, it has been suggested that the maxillary central incisors and canines should be placed at relatively similar level, with incisal edge of the lateral incisors placed or positioned 1 to 1.5 mm higher.^[31,33]^

These studies however did not establish the esthetic impact of variations in the vertical position of the maxillary lateral incisor clearly.^[31]^ This was the literature gap that the current study sought to close. The smile attractiveness is different from culture to culture, and from country to country. However, the attractiveness criteria in Saudi Arabia was not yet investigated. Moreover, even though smile esthetic should be the concern of all dental specialists and not just the orthodontics, no studies evaluated the perceptions of prosthodontics and esthetic and restorative dentists. Each specialty studied their smile esthetic from their prospective. No study like the current 1 compared between all the specialties, laypeople and the students.

For the current investigation, only the mouth area was used to limit the effect of confounders.^[26]^ Canine vertical position affects the smile attractiveness as it affects the smile arc. Scarce studies focused on the position and its effect on smile attractiveness. Smile arc is also affected by the central incisors’ positions and their relation to each other. Most of the studies focused on the maxillary incisors position.^[27,29]^ According to a systematic review, 8 articles exist today that focused on incisors position.^[13]^ One study investigated the vertical position of the canine and the difference in the smile perception between laypeople and orthodontist.^[30]^

This study was designed to close these gaps in the literature. Results were insightful and should be taken into consideration in orthodontic, prosthodontics, and cosmetic dentistry practice, especially in Saudi Arabia. Results of the study confirmed what past researchers did, that the orthodontist was more critical in their assessment. Unique to this study set in the Saudi Arabian context however is the finding that specialists from other dental fields are just like the laypersons in their assessment of the canine and incisors vertical positions. They are not as thorough as the orthodontists. Results also showed that the Saudi laypeople were more sensitive to the vertical positions of the canine, in contrast to the American population, who was more sensitive to the asymmetric changes in the incisors, as reported in previous studies.^[27]^

Overall, these findings can be explained that in general, specialists know more about dental esthetics compared to laypeople and students, who do not have enough knowledge about the smile esthetic. Naturally, with greater knowledge comes lower levels of tolerance. Laypeople can tolerate up to 1 mm differences in incisor position. However, one of the limitations of this study is there was only 1 patient included in the study, which we need to include more patients, with different tooth shapes, color, etc in the future.

In the Saudi Arabian context as well, laypeople were more sensitive to the changes in the canine vertical position (.5 mm –1 mm) than the orthodontist (1 mm) and dentist from other specialties. Undergraduate students are more sensitive to the changes in both canine and incisors. This may suggest that the age of the students made them still unknowledgeable about the ideal smile in the dental context.^[34–42]^

With regard to the best incisor positions, only the laypeople and prosthodontics liked the original picture. The rest of the participant groups liked +0.5 mm which accentuate the smile curve and make it follow the lower lip line. In relation to the worst incisor position, all groups did not prefer the minus 1.5 reversed smile. This is in accordance with what is found in the literature.^[25,26]^ As for the best canine vertical position, all groups preferred the original position where canine set at zero (at the level of the incisal plane). Lastly, with regard to the worst canine position, they all disliked the minus 1.5.

In conclusion, results confirmed past findings that orthodontists are in general more critical. However, present findings showed that they are not just more critical compared to laypersons, but also to other dental specialists. Marked differences between the Saudi Arabian population on their perceptions on what makes a smile attractive as well, compared to the Western population. These findings can certainly be of utility in the esthetic dentistry field and several dental issues are only noticeable by specialists, and not by majority of the public. Moreover, multiple dental issues will not require any dental treatments if will not cause any harm and not noticeable. However, further research on these dental design parameters and their specific hierarchy of influence on smile attractiveness in the Saudi Arabian context remain necessary.

## 5 Clinical implications

The vertical position of the maxillary central incisors can significantly affect the perceptions of esthetics among the Saudi Arabian population. However, the orthodontists were more critical in their assessment compared to other specialists and compared to the laypeople. Past studies often showed that dentists and laypeople did not perceive the canine and incisors in vertical positions similarly, but failed to account for differences between orthodontists and other dental specialists, which mean several dental issues are only noticeable by specialists (orthodontists and other specialists) and not noticeable by majority of the public. The current findings closed that gap in the context of Saudi Arabia. Understanding these differences can lead to better and more satisfying orthodontic and prosthodontics diagnosis and treatment planning.

## Author contributions

Study design: Dr. WB, ZB. Recruitment of samples, obtaining X rays: Dr. WB, ZB. Radiographic analysis and measurements: Dr. WB, ZN. Interpretation of data and statistical analysis: Dr. WB, ZN. Manuscript preparation: Dr. WB, ZN. Manuscript review and approval: Dr. WB, ZB, ZN. Accountability for author's contributions: Dr. WB, ZB, ZN. The author(s) read and approved the final manuscript.

**Conceptualization:** Walaa A. Babeer, Zuhair T. Bakhsh, Zuhair S Natto.

**Data curation:** Walaa A. Babeer, Zuhair T. Bakhsh, Zuhair S Natto.

**Formal analysis:** Walaa A. Babeer, Zuhair S Natto.

**Methodology:** Walaa A. Babeer, Zuhair T. Bakhsh.

**Project administration:** Zuhair S Natto.

**Software:** Walaa A. Babeer, Zuhair T. Bakhsh.

**Supervision:** Zuhair S Natto.

**Validation:** Walaa A. Babeer, Zuhair T. Bakhsh.

**Writing – original draft:** Walaa A. Babeer, Zuhair T. Bakhsh, Zuhair S Natto.

**Writing – review & editing:** Walaa A. Babeer, Zuhair S Natto.
